# Fecal metabolome of the Hadza hunter-gatherers: a host-microbiome integrative view

**DOI:** 10.1038/srep32826

**Published:** 2016-09-14

**Authors:** Silvia Turroni, Jessica Fiori, Simone Rampelli, Stephanie L. Schnorr, Clarissa Consolandi, Monica Barone, Elena Biagi, Flaminia Fanelli, Marco Mezzullo, Alyssa N. Crittenden, Amanda G. Henry, Patrizia Brigidi, Marco Candela

**Affiliations:** 1 Department of Pharmacy and Biotechnology, University of Bologna, Bologna 40126, Italy; 2 Laboratories of Molecular Anthropology and Microbiome Research, University of Oklahoma, Norman, OK 73019, USA; 3 Institute of Biomedical Technologies, Italian National Research Council, Segrate, Milan 20090, Italy; 4 Endocrinology Unit, Department of Medical and Surgical Sciences and Center for Applied Biomedical Research, University of Bologna – S. Orsola-Malpighi Hospital, Bologna 40138, Italy; 5 Metabolism, Anthropometry, and Nutrition Laboratory, Department of Anthropology, University of Nevada, Las Vegas, NV 89154-5003, USA; 6 Plant Foods in Hominin Dietary Ecology Research Group, Max Planck Institute for Evolutionary Anthropology, Leipzig 04103, Germany

## Abstract

The recent characterization of the gut microbiome of traditional rural and foraging societies allowed us to appreciate the essential co-adaptive role of the microbiome in complementing our physiology, opening up significant questions on how the microbiota changes that have occurred in industrialized urban populations may have altered the microbiota-host co-metabolic network, contributing to the growing list of Western diseases. Here, we applied a targeted metabolomics approach to profile the fecal metabolome of the Hadza of Tanzania, one of the world’s few remaining foraging populations, and compared them to the profiles of urban living Italians, as representative of people in the post-industrialized West. Data analysis shows that during the rainy season, when the diet is primarily plant-based, Hadza are characterized by a distinctive enrichment in hexoses, glycerophospholipids, sphingolipids, and acylcarnitines, while deplete in the most common natural amino acids and derivatives. Complementary to the documented unique metagenomic features of their gut microbiome, our findings on the Hadza metabolome lend support to the notion of an alternate microbiome configuration befitting of a nomadic forager lifestyle, which helps maintain metabolic homeostasis through an overall scarcity of inflammatory factors, which are instead highly represented in the Italian metabolome.

The recent characterization of the gut microbiome of the Hadza hunter-gatherers of Tanzania^1^,^2^, one of the best populations in which to ask evolutionary questions, allowed us to appreciate the specific adaptive versatility of the microbiome to different modes of life and subsistence of traditional populations, opening up, however, significant questions on whether and how microbiota alterations occurring in industrialized urban populations may have contributed to the growing list of Western diseases^3^. The documented shifts in microbiota composition in Western populations, and especially the loss of co-evolved microbial species^1^,^2^,^3^,^4^,^5^,^6^, could in fact have meant the loss of a primary asset for the human host in industrialized societies, leading to important changes in the metabolite repertoire at our disposal, influencing multiple metabolic and immunological pathways and ultimately altering human biological fitness.

A large array of small molecules are generated in the gastrointestinal tract, as a result of the interactive chemical communication network between the host and its resident microbiota. Many of these metabolites play critical biological roles not confined to the intestine but physiologically connected to distant organs, including the liver, skeletal muscles and brain, which strongly contribute to the host metabolic phenotype and hence to the state of health^7^. However, to date, we are far from understanding how human metabolism integrates the activities of the intestinal microbiota, and we do not know whether populations with different lifestyles differ in microbial metabolite production and how these differences may impact the microbiota-host meta-metabolic network.

In an attempt to bridge this gap, here we applied a targeted approach to profile the fecal metabolome of two populations exemplifying two distinct and opposing subsistence regimes along the spectrum of human life-ways: the Hadza of Tanzania, who forage for the bulk of their diet and live in the absence of agriculture, and urban living Italians, as representative of people in the post-industrialized West. The Hadza are one of the world’s few remaining hunting and gathering communities, living in small mobile camps around the shores of Lake Eyasi in Northern Tanzania^8^. As such, they represent a unique opportunity to approximate the microbiota-host configurations arising from a foraging subsistence strategy, similar to that of ancient humans, which has accompanied over 90% of our evolutionary history^9^. In order to account for the contribution of intestinal bacteria in shaping the metabolome of Hadza and Italians, we focused on fecal metabolites correlated with the respective gut microbiota profiles. The results were interpreted in the light of recent findings arising from metagenome sequencing of the Hadza gut microbiota in a host-microbiome integrative view.

## Results

We recently outlined the peculiarities of the Hadza gut microbiome, which led us to uncover a unique structural and functional configuration that aligns with their foraging lifestyle, as well as to appreciate the essential co-adaptive role of the gut microbiome in complementing human physiology^1^,^2^.

To improve our understanding of the functional aspects of the microbiota-host interactions in regards to disparate modes of human life and subsistence, we applied a targeted metabolomics approach to fecal samples from 17 Hadza (age: 15–65 years) and 12 Italians (age: 23–40 years). In particular, as a sub-cohort from Schnorr *et al*.^2^, Hadza stools had been collected in 2013 during the rainy season, when the Hadza diet is primarily based on plant foods. For Italian controls, new stools were collected for this study from the same Italian adults recruited as a Western cohort in Schnorr *et al*.^2^. Through our metabolomics approach, based on a phenotyping kit combining a flow injection/LC-MS/MS assay to cover key metabolites from main metabolic pathways (Absolute*IDQ* p180 kit, BIOCRATES), we detected and quantified 186 out of the 188 targeted metabolites, failing to recover nitrotyrosine and phosphatidylcholine acyl-alkyl C42:4 (PC ae C42:4) (see Supplementary Table S1). In order to focus on metabolites possibly contributed by intestinal bacteria or attributable to microbial activity in the gut, we sought correlations between metabolite levels and relative abundances of gut microbiota genera. To this aim, we performed the 16S rRNA next-generation sequencing of the new fecal samples from Italians, and recovered the Hadza microbiome data from our previously published paper^2^. As many as 180 metabolites are found to be significantly correlated (positively or negatively; *P* < 0.05, Kendall tau correlation test) with at least one genus of the gut microbiota. Compounds that were filtered out for lack of correlations include alpha-amino acid components of Lys (alpha-aminoadipic acid), Pro (cis- and trans-OH-Pro) and Gly (sarcosine) metabolism, as well as dopamine and its precursor, DOPA.

**Figure 1:**
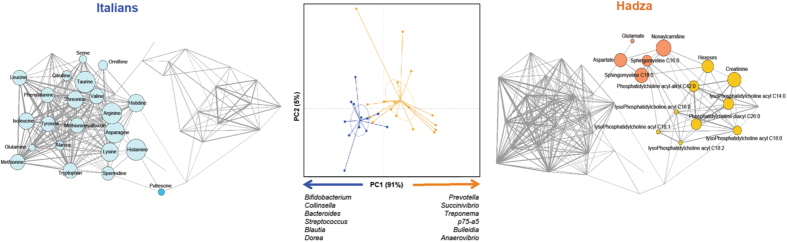
Fecal metabolome difference between Hadza and Italians. At the center, Principal Component Analysis (PCA) of Euclidean distances between metabolic profiles of the fecal extracts from 17 Hadza (orange) and 12 Italians (blue). *P* = 7 × 10^−5^, permutation test with pseudo F ratios. Genera of the gut microbiota significantly correlated to PC1 (*P* < 0.05, Kendall tau correlation test) are displayed at the bottom. Microbial genera were filtered for those with >0.1% of relative abundance in at least 30% of subjects. At the sides, Wiggum plots indicate the pattern of variation of the four identified metabolic co-abundance groups (CAGs) in Hadza (right) and Italians (left). Each node represents a fecal metabolite and its dimension is proportional to the over-abundance relative to background. Connections between nodes indicate positive and significant Kendall correlations between metabolites (*P* < 0.05). Line thickness is proportional to correlation strength. Only fecal metabolites significantly correlated (positively or negatively) with at least one genus of the gut microbiota (*P* < 0.05, Kendall tau correlation test) were considered. For Wiggum plots, correlated metabolites were further filtered for those with >0.05% of relative abundance in at least 20% of subjects. See also Supplementary Fig. S4.

Principal Component Analysis (PCA) of the relative abundance profiles of microbiota-related metabolites shows separation by population (*P* = 7 × 10^−5^, permutation test with pseudo F ratios; Fig. 1), as the PCA of the respective genus-level gut microbiota profiles does (*P* = 0.0001; see Supplementary Fig. S1). Given that PC1 explains the vast majority of variance within the metabolic dataset, we used PC1 to look for correlations with microbial genera. As expected, PC1 coordinate-related genera are among those already identified as discriminatory for the Hadza in Schnorr *et al*.^2^. In particular, *Prevotella*, *Succinivibrio*, *Treponema* and *Bulleidia* are positively correlated to the axis (*P* ≤ 004, Kendall tau correlation test), confirming their association with discriminatory metabolite production in Hadza. However, *Bifidobacterium*, *Bacteroides*, *Blautia* and *Dorea* are correlated with negative values of PC1 coordinates (*P* ≤ 0.02), corroborating the earlier findings that traditionally considered mutualistic microbiota found amongst Western populations are actually underrepresented in the Hadza microbiota. Unlike what was documented by Schnorr *et al*.^2^ in the Hadza gut microbiota taxonomic structure, we found no evidence of a sex-related divergence in the metabolome (*P* > 0.05, permutation test with pseudo F ratios; see Supplementary Fig. S2). However, it should be noted that the low number of women in the sub-cohort used in the present study (5 females vs 12 males) may have precluded us from detecting significant gender differences. In order to control for age contribution to the metabolome variation, a multiple regression analysis was carried out. According to our data, age was found to account for less than 10% of variance of the metabolome response (see Supplementary Methods, Supplementary Fig. S3 and Supplementary Table S2), suggesting a minor effect of age on the metabolite repertoire across our study cohort.

**Figure 2:**
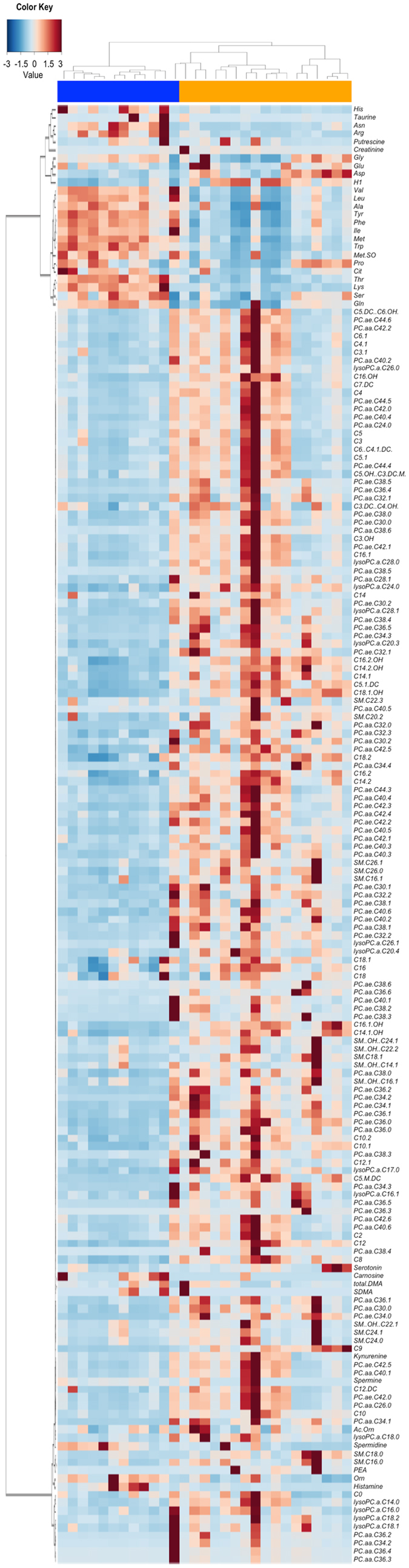
Fecal metabolic profiles of Hadza and Italians. Heat map shows the relative abundance of fecal metabolites in Hadza (orange) and Italians (blue). Fecal metabolites were filtered for significant correlation (positive or negative) with at least one genus of the gut microbiota (*P* < 0.05, Kendall tau correlation test). Hierarchical clustering was performed using the Pearson distance measure and Ward linkage method. *P* = 3 × 10^−7^, Fisher’s exact test. For the full name of metabolites, please see Supplementary Table S1.

Hierarchical clustering based on Pearson correlation coefficients of the fecal metabolic profiles confirms the separation between Hadza and Italian metabolome (*P* = 3 × 10^−7^, Fisher’s exact test; Fig. 2). Two main metabolite clusters are visible, and each indicates further subgroup structure. The first major cluster includes the most common natural amino acids (except for Cys, which was undetectable by this metabolomics approach) whose abundances are generally greater in Italians (cumulative relative abundance, 78.1% vs Hadza, 43.8%), amino acid derivatives (including citrulline, Met-SO, putrescine and taurine), creatinine and hexoses. Of note, hexoses are far more represented in the Hadza fecal metabolome (50.5% vs Italians, 16.3%). The second cluster includes all targeted acylcarnitines, sphingolipids and glycerophospholipids, which are also largely overabundant in the Hadza (cumulative relative abundance in Hadza vs Italians, 0.5% vs 0.2%, 0.2% vs 0.06%, and 1.1% vs 0.6%, respectively), together with some amino acid derivatives, spermine and its precursor spermidine. According to a Random Forests analysis^10^, eight metabolites are highly discriminatory between Hadza and Italians (cross-validation error = 0.034 ± 0.000), 7 over-represented in the first (PC aa C32:0, PC aa C36:5, PC ae C34:0, C5-M-DC, C9, C16:2-OH, C18:1-OH) and 1 in the latter (Tyr), confirming the existence of distinct metabolic signatures for Hadza and Italians.

To explore the patterns of metabolome variation between populations, we established co-abundance associations of metabolites and then clustered correlated metabolites into four co-abundance groups (CAGs; *P* < 0.05, permutation multivariate analysis of variance; see Supplementary Fig. S4)^11^. The difference in the CAG profiles for Hadza and Italians confirms the separation between their metabolome structures as previously seen in the clustering analysis (Fig. 1). Hadza are indeed characterized by an enrichment in hexoses, phosphatidylcholines and sphingomyelins, along with nonaylcarnitine, creatinine, aspartate and glutamate. On the other hand, they are overall depleted in the most common natural amino acids and derivatives, as well as the polyamines putrescine and spermidine.

## Discussion

The Hadza metabolites measured in our study were present in the gut when their dominant foods were tubers, baobab, and honey, along with consumption of some game meat, but not on a daily basis. The rainy seasons, including the intermittent period from January to March that encompassed our sampling window, span half of the year, and so a snapshot of the metabolome during this time of year, as determined in the present study, is relevant for characterizing metabolites produced while Hadza consume primarily a plant-based diet.

The Hadza and Italians possess not only distinct gut microbiota profiles but also divergent metabolomes, reflecting different prioritization of functional pathways among the microbial communities. Such divergence is expressed mainly in different proportions of hexoses (Hadza vs Italians, 50.5% vs 16.3%), sphingolipids (0.2% vs 0.06%), and glycerophospholipids (1.1% vs 0.6%), as well as amino acids and biogenic amines (47.7% vs 82.8%). It should be noted that, through our targeted metabolomics approach that covers 188 key metabolites from main metabolic pathways, we failed to detect nitrotyrosine and phosphatidylcholine acyl-alkyl C42:4. While no information on the biological role of this last metabolite is known or available to the authors, except for a recent associations with single nucleotide polymorphisms in FADS1-3 loci (encoding fatty acid desaturases)^12^, increased nitrotyrosine levels are generally recognized as markers of inflammation/oxidative stress, and so far detected in inflammatory bowel disease and other conditions of increased intestinal permeability^13^,^14^. Differently, in healthy subjects, no or little presence of nitrotyrosine has been demonstrated along the intestine^14^,^15^,^16^. Considering that no Hadza camp members appeared sick or to have overt infections during collection of fecal samples, the absence of nitrotyrosine in their stools as well as in those of Italians is not really so surprising.

To date, only Gomez *et al*.^4^ have explored the stool metabolome of a hunter-gatherer population, the BaAka Pygmies from Central African Republic, demonstrating a dominance of lipids (40.8% of all metabolites), followed by carbohydrates (19%), sterols (18.9%), phosphates (8%), organic acids (7.9%), amino acids and amines (3.3%), and bile acids (1.7%). Unfortunately, the metabolomic approaches used are different, based on GC/MS and a custom-built library of metabolites for Gomez *et al*.^4^, and a metabolic phenotyping kit with a combined flow injection/LC-MS/MS assay for the present study, making it difficult to make a direct comparison between the metabolic profiles of Hadza and BaAka Pygmies. Moreover, differently from our study, the data from Gomez *et al*.^4^ were not placed in context with any other population, making impossible any evolutionary, lifestyle-related assessment.

As revealed from the metagenomics analysis of the Hadza microbiome^1^ and recently confirmed across geography^17^, individuals from remote rural locations in Africa and Latin America, including the Hadza hunter-gatherers, have an extremely high abundance and diversity of carbohydrate-active enzymes (CAZymes), as compared to urban Westernized populations. This may represent an adaptation of their gut microbiota to the specific indigenous diets that, in the case of the Hadza especially during the rainy season at the time of sample collections, comprise mostly unrefined plant foods containing high amounts of indigestible polysaccharides that most likely reach the colon^2^. The increased levels of hexoses in the feces of the Hadza may thus be a direct indicator of activity of the highly enriched microbial CAZyme repertoire they possess, releasing free monosaccharide units from complex polysaccharides, exemplary of the extensive role of the gut microbiota in fiber degradation. Perhaps through enhanced CAZyme activity, it is possible for Hadza microbiota to meet or even exceed the nutritional demands of the enteric ecosystem and therefore provision the host as well. On the other hand, we cannot exclude that such hexoses result from anabolic processes, i.e. gluconeogenesis and the pentose phosphate pathway in its non-oxidative phase. In support of this, the Hadza gut microbiome is significantly enriched in genes coding for phosphoenolpyruvate carboxykinase, a key regulatory enzyme driving gluconeogenesis, malate dehydrogenase, and intermediary enzymes in the pentose phosphate pathway^1^. In the same way, the high representation of genes involved in alanine, aspartate and glutamate metabolism, as detected in the Hadza microbiome^1^, could allow the conversion of these excess glucogenic amino acids, particularly abundant in baobab seed pulp that Hadza habitually consume^18^,^19^, and abundantly found in their feces as demonstrated in the present study, to glucose. Finally, another major contribution to the generation of hexoses could result from the enrichment in microbial gene assignments for propionyl-CoA carboxylase^1^, which ultimately leads to formation of several gluconeogenic intermediates.

The Hadza fecal metabolome was also characterized by an overall surplus of sphingolipids, specifically C16:0 and C18:0 sphingomyelins (SMs). From a microbiological perspective, such sphingolipids could be imparted directly by the cell membrane of some intestinal bacteria. Though far from being an exhaustive list, some bacterial species have been proven to contain sphingolipids, especially in the Bacteroidetes phylum^20^,^21^, which is particularly enriched in the Hadza gut microbiota^2^. Alternatively, some microbiota express and secrete enzymes with sphingolipid-metabolizing capability^22^,^23^, or even influence the activity of the human alkaline sphingomyelinase, a key enzyme catalyzing the first step in the intestinal SM degradation^24^,^25^. Rampelli *et al*.^1^ showed that genes involved in sphingolipid metabolism are poorly represented in the Hadza metagenome, suggesting a low SM-catabolizing capacity for Hadza gut microbes. In addition to being introduced to the gut through the diet^26^, SMs are ubiquitous structural components of mammalian cell membranes and specifically abundant in the microvillar membrane of intestinal epithelial cells^27^. In the context of the gut microbiota-host environment, sphingolipids are largely of host origin^22^. The metabolism of dietary and membrane SMs is dependent on alkaline sphingomyelinase activity, which is also present in human bile, with taurocholate and taurochenodeoxycholate bile salts being the most effective stimulators^28^. The high amount of SMs in the Hadza feces compared to Italians could be partly attributable to a difference in human alkaline sphingomyelinase activity, which is consistent with observations that high-fiber intake reduces bile acids excretion^29^ while a typical high-fat Western diet stimulates cholecystokinin production and induces a shift of the bile acid pool from glycine to taurine-conjugation^30^. Still, despite sparse and contrasting information, SMs are known to trigger anti-inflammatory and anti-proliferative cascades, mainly through their metabolic by-products, including ceramide, sphingosine, and their phosphorylated counterparts, but also by themselves^27^. SMs have been indeed reported to inhibit the PLA2 activity, like several anti-inflammatory drugs, thereby protecting phosphatidylcholines (PCs) from premature or excessive hydrolysis to arachidonic acid and lysophosphatidylcholines (lysoPCs), which are in turn precursors of PGE2, leukotrienes and lysophosphatidic acid, which act as potent stimulators of cell migration and inflammation^31^. Furthermore, SMs can inhibit cholesterol absorption, potentially influencing risk factors for metabolic disorders^32^.

Bearing in mind the above considerations on a possible difference in human alkaline sphingomyelinase activity between Hadza and Italians, and considering that this enzyme also hydrolyses other choline phospholipids, including the glycerophospholipids PCs and lysoPCs^33^, it is not surprising that even these metabolites are extremely enriched in the Hadza stools. Phospholipids in the feces can be derived from the diet, bile, shed epithelial cells, and bacterial cells. First identified in egg yolk and predominantly found in milk, meat, and fish^34^, PCs are actually present throughout the vegetable kingdom and have been so far detected in a number of plants, including seeds and tubers, mainly belonging to the legume family, Fabaceae^35^. This is particularly relevant as tubers, specifically from Fabaceae and Convolvulaceae plant families, represent an incredibly important, consistently available food resource for the Hadza, and are exploited year round^36^. However, the diet is unlikely to contribute a dominant fraction to the fecal phospholipid pool. Instead, dietary PCs are generally hydrolyzed and absorbed by the small intestine^37^, even though this may not be true for plant phospholipids trapped in a cellulose matrix. On the other hand, PC is the major phospholipid in bile^38^, and up to 90% of intestinal mucus consists of PCs and lysoPCs, which are largely responsible for mucus hydrophobicity^39^,^40^. Although they are a typical eukaryotic membrane phospholipid, PCs can be also found in some bacteria, particularly those closely interacting with eukaryotic hosts in a symbiotic or pathogenic relationship^41^,^42^. To date, most PC-containing species are known to belong to Proteobacteria and Spirochaetes, including *Treponema*^43^, which are consistently found within the gut microbiota of all traditional rural or foraging societies studied to date and absent from that of urban-industrial populations^2^,^4^,^5^. Specifically, *Treponema* genomes harbor homologues of CDP-choline pathway for PC biosynthesis, likely using choline as a building block in a camouflage strategy, which is released by eukaryotic partners; whether human, animal or plant host^43^. Interestingly, a decreased relative abundance of several PC species has been found in the feces of mice fed a high-fat diet^44^, and similar results have been obtained regarding the PC and lysoPC content of mucosal aliquots from patients with ulcerative colitis (UC)^39^. It is possible that a defective PC layer exerts an impaired barrier function, allowing bacterial binding to the epithelium and contributing to the development of inflammation typical of the so-called Western diseases. When orally administered to patients with UC, modified release PCs are shown to result in an overall decreased inflammatory activity, possibly helping to rebuild the mucus structure and density, as well as intervening in signaling networks ultimately leading to inhibition of mucosal inflammation^45^. PC supplementation reportedly leads to significant improvements of other inflammatory conditions that are increasingly prevalent in Western populations, such as obesity and its comorbidities, including ischemia, non-alcoholic fatty liver disease and cardiovascular disease^46^,^47^,^48^. Accordingly, the large quantities of non-hydrolyzed PCs and lysoPCs detected in the Hadza fecal metabolome, and as supported by the reduced capacity for glycerophospholipid metabolism found in their metagenome^1^, could have important health implications at both enteric and somatic level, protecting the host from a variety of diseases.

Additionally, we found an overall lower abundance of amino acids and derivatives in the Hadza fecal metabolome compared to Italians, which may suggest differences between the two populations at various levels, including protein consumption patterns, degradation of dietary proteins or of intestinal mucins, and variation in intestinal absorption or secretion of amino acids. Rampelli *et al*.^1^ showed that the Hadza gut microbiome is equipped for branched-chain amino acid (BCAA) degradation and aromatic amino acid biosynthesis, whereas the Italian metagenome is enriched in protein metabolic pathways mainly devoted to BCAA biosynthesis. In the context of a high-fat diet, an overabundance of circulating BCAA has been implicated in the development of insulin resistance, diabetes, and related complications^49^. In addition, BCAA and other amino acids are often found to be increased in fecal extracts or mucosal biopsies from patients with inflammatory bowel disease (IBD), probably because of the large energy demand of inflammatory conditions or due to protein losing enteropathy^50^,^51^. Based on these observations, an amino acid-poor metabolic profile, as that detected in the Hadza feces, could then be indicative of an intestinal ecosystem somehow less prone to develop or sustain inflammation signaling.

In summary, the characterization of the metabolic phenotype of the Hadza during the rainy season, when their diet is primarily based on plant foods, allowed us to make a step toward deciphering the intricate host-microbe relationships that regulate human biology and have contributed to our evolutionary history. In parallel to the peculiar features of their microbiota and microbiome, the Hadza fecal metabolome showed a unique enrichment in metabolite classes that may have an impact on human health. Our findings lend support to the notion that the enteric ecosystem co-evolved in the ancient selective environments of our presiding forager legacy, thus complementing human physiology in present day hunter-gatherers. In addition to the abundance of hexoses (simple sugars), that seems to be indicative of a diet rich in microbiota-accessible carbohydrates (MAC)^3^, the high presence of sphingolipids and glycerophospholipids, together with low levels of amino acids, suggests a robustly healthy gut metabolic profile that is specifically poor in factors known to trigger or contribute to the typical inflammation-based Western diseases. The high-MAC diet, as that of the Hadza and other traditionally living humans, may then be uniquely permissive of a diverse community of gut microbiota that also demonstrates a functional specificity aligned with preserving local and systemic health and mitigating disease risks^52^. However, since metrics of health, such as immunological parameters, were not measured in the present study, whether the Hadza have actually lower inflammation than the Italian cohort, still remains to be investigated. Anthropometric data for Hadza exists in Pontzer *et al*.^53^ and curiously, they have very low resting metabolic rate compared to the Tsimane, a population of forager-horticulturalists living in Amazon Bolivia^54^, which would indirectly suggest that Hadza have low immune stress, likely related to a low pathogen burden. Finally, it should be mentioned that genetic heritage and factors related to aspects of lifestyle, other than diet (i.e. climate, geography, presence of parasites, etc.), already known to impact the microbiome, may likewise contribute to explain the metabolite differentiation between Hadza and Italians. Further studies aimed at unravelling the complex microbiota-host meta-metabolic network in ancient and modern populations are thus needed to address significant questions about human biology throughout evolution.

## Methods

### Sample collection and preparation

Fecal aliquots of the Hadza for this study came from the same original samples from Schnorr *et al*.^2^, and represent a sub-cohort of 17 Hadza volunteers from the Dedauko and Sengele camps in Northern Tanzania (age: 15–65 years; mean, 34). These stools had been collected in 2013, during the rainy season, when the dominant foods consumed by Hadza were tubers, baobab, and honey, along with consumption of some game meat, but not on a daily basis. Italian adults, living in and around Bologna, recruited as a Western cohort in Schnorr *et al*.^2^, were recalled to provide fecal specimens; as many as 12 subjects (age: 23–40 years; mean, 33) provided the samples. Informed consent was obtained from all the subjects enrolled. Since Hadza are non-literate, verbal consent was obtained by those who agreed to participate, and this was documented by a separate witness. In the case of young Hadza, we obtained verbal assent from the youths and verbal consent from the parents, which was again documented by a separate witness. All work was approved by the University of Leipzig Ethik-kommission review board (reference number 164-12-21052012), Tanzanian Commission for Science and Technology (COSTECH, permit number 2012-315-NA-2000-80), and the Tanzanian National Institute for Medical Research (NIMR). Methods were carried out in accordance with the approved guidelines.

For metabolite analysis, about 200 mg of homogenized feces were extracted by the addition of three equivalents (w/v) of methanol followed by vortex-mixing (3 sec) and sonication for 10 minutes. Fecal extracts were recovered by centrifugation at 14,000 rpm for 10 minutes at 4 °C, filtered through 0.22-μm PES membranes and then stored at −80 °C until analysis.

### Targeted metabolomics

A targeted quantitative metabolome approach was applied using the Absolute*IDQ* p180 Kit (BIOCRATES Life Sciences AG, Innsbruck, Austria) that allows the quantification of up to 188 key metabolites from main metabolic pathways, including acylcarnitines, amino acids, biogenic amines, hexoses, sphingolipids and glycerophospholipids (see Supplementary Table S1), in multiple biological matrices, including feces. However, since the Absolute*IDQ* kit has been validated for plasma, an optimization of the extraction solvent for fecal samples was necessary. Methanol, phosphate buffer 10 nM, pH 7.5 (PBS) and a mixture of methanol/PBS 70:30 were tested by analyzing replicates (n = 3) and by comparing yield for all metabolite classes (CV% within 25). The results showed that there is not a solvent of choice for all the analytes, however for the majority of the metabolites (acylcarnitines, PCs, lysoPCs, SMs) methanol gave the best overall results (see Supplementary Fig. S5). For amino acids and hexoses, the mixture methanol/PBS and PBS resulted in higher yield but they were concentrated enough, not requiring higher sensibility for this kind of samples.

After extraction, fecal samples were analyzed according to the manufacturer’s instructions. Briefly, based on PITC (phenylisothiocyanate)-derivatization in the presence of isotopically labelled internal standards, the assay combines flow injection (FIA) and LC-MS/MS analysis. LC-MS/MS and FIA plate were run on a Series 200 HPLC (PerkinElmer, Waltham, MA) coupled with a 4000QTrap mass spectrometer operated in triple quadruple mode (AB-Sciex, Toronto, Canada). Data processing and elaboration were performed by Analyst 1.6.3.

### Illumina MiSeq sequencing and data analysis

After isolation of microbial DNA from Italian feces using repeated bead beating^55^ as in Schnorr *et al*.^2^, the V3-V4 hypervariable region of the 16S rRNA gene was amplified using the 341F and 805R primers with added Illumina adapter overhang sequences as previously reported^56^. PCR reactions were cleaned up with Agencourt AMPure XP magnetic beads (Beckman Coulter, Brea, CA). Attachment of dual indices and Illumina sequencing adapters was performed by limited-cycle PCR using Nextera technology. After subsequent clean-up as described above, libraries were normalized to 4 nM and pooled. The sample pool was denatured with 0.2 N NaOH and diluted to a final concentration of 6 pM with a 20% PhiX control. Sequencing was performed on Illumina MiSeq platform using a 2 × 300 bp paired end protocol, according to the manufacturer’s instructions (Illumina, San Diego, CA).

Raw sequences were processed using a pipeline combining PANDAseq^57^ and QIIME^58^. Quality filtered reads were clustered into OTUs at 97% similarity threshold using UCLUST^59^. Taxonomy assignment was conducted using the RDP classifier against the Greengenes database (May 2013 release). Pyrosequencing reads for the Hadza^2^ were re-assigned as well. All singleton OTUs were discarded. Sequencing reads were deposited in the National Center for Biotechnology Information Sequence Read Archive (NCBI SRA; BioProject ID PRJNA340060).

## Statistics

All statistical analysis was performed in R 3.1.3. The Kendall tau correlation test between the relative abundance of fecal metabolites and bacterial genera was achieved using stats package. Genera of the gut microbiota were filtered for those with >0.1% of relative abundance in at least 30% of subjects. Euclidean distances between fecal metabolic or microbiota profiles were used for Principal Component Analysis (PCA) and plotted by the vegan package. Data separation in PCA space was tested using a permutation test with pseudo F ratios (function adonis in vegan package). Separation along PC1 was verified by Wilcoxon rank-sum test. Heat map analysis was performed using the made4 package. Metabolite co-abundance groups (CAGs) were determined as previously described^11^. Metabolites significantly correlated to the genus-level gut microbiota profiles were further filtered for those with >0.05% of relative abundance in at least 20% of subjects. Wiggum plots were created using Cytoscape 3.2.1. Discriminatory metabolites between Hadza and Italians were identified using the Random Forests machine learning algorithm^10^. Briefly, Random Forests is a powerful classifier that identifies the best subset of features (here, relative metabolite abundance) at discriminating between categories (Hadza vs Italian fecal metabolome). To explore the influence of age on the metabolome profile from Hadza and Italians, a multiple regression analysis was used (please see Supplementary Methods). Briefly, we constructed a model taking into account as explanatory variables age, sex and microbiome taxonomic composition, and tested the influence of each factor on the measured metabolome response. Where necessary, P values were corrected for multiple comparisons using the Benjamini-Hochberg method. *P* < 0.05 was considered as statistically significant.

## Additional Information

**How to cite this article**: Turroni, S. *et al*. Fecal metabolome of the Hadza hunter-gatherers: a host-microbiome integrative view. *Sci. Rep*. **6**, 32826; doi: 10.1038/srep32826 (2016).

## Supplementary Material

Supplementary Information
