# Supplementary material for: Fecal metabolome of the Hadza hunter-gatherers: a host-microbiome integrative view
Source: Sci Rep. 2016 Sep 14;6:32826. doi: 10.1038/srep32826 (PMC5021991; doi:10.1038/srep32826)
Supplement: Supplementary Information [file srep32826-s1.pdf]

Fecal metabolome of the Hadza hunter-gatherers: a host-microbiome integrative view

Silvia Turroni, Jessica Fiori, Simone Rampelli, Stephanie L. Schnorr, Clarissa Consolandi, Monica Barone, Elena Biagi, Flaminia Fanelli, Marco Mezzullo, Alyssa N. Crittenden, Amanda G. Henry, Patrizia Brigidi & Marco Candela

Supplementary Methods

To explore the influence of age, sex, and microbiome taxonomic composition on the metabolome profile from Hadza and Italian stool samples, we used a multiple regression analysis. In doing so, we can determine which of the explanatory variables best explains the measured metabolome response. Due to the high dimensionality of the metabolome and taxonomic datasets, it was necessary to first perform principle components analysis and run the regression using site scores for the new eigenvectors. For the metabolome data, >90% of the variance was explained by PC1, and for the taxonomic data, PC1-PC6 cumulatively accounted for >90% of variance. Therefore, the dimensions of the data were reduced sufficiently to obtain a stable model. We checked various diagnostics of model validity and stability, as well as for collinearity of the explanatory variables. In doing so, we discovered one instance of collinearity between PC1 of the taxonomy and population group assignment (Hadza or Italian; see Supplementary Fig. S3A online). Therefore, the final model was run without the explanatory variable for group. We fitted the model using the 'lm' function of the statistic package in R (version 3.3.1). Distribution of the residuals and analysis of normal Q-Q plot were not suggestive of deviations from assumptions of normality and homoscedasticity (see Supplementary Fig. S3B online). Variance inflation factors (VIF) to check for collinearity and DFBetas as well as Cook's distance to check for overly influential cases did not reveal any problems.

The final model formula was the following:

lm(metabMDS1 ~ taxMDS1+taxMDS2+taxMDS3+taxMDS4+taxMDS5+taxMDS6+Age+Sex)

To test the significance of the predictors as a whole (the model), we compared the fit of the model to that of the null comprising only the intercept, and find that the model is an ok fit and slightly significant (p=0.042; df=20; F=2.57; Adjusted R-squared=0.31) (see Supplementary Table S2 online). To test the influence of each factor, we evaluated the residual sum of squares for effect size and found that taxMDS1 explains 34% of the variance of the metabolome response, while the remaining variables account for less than 10% of variance. Plotting this effect, we see that the

variance in the metabolome MDS1 and the taxonomy MDS1 are inversely associated (see Supplementary Fig. S3C online). This indicates overall that the metabolome profile is, not surprisingly, best explained by the variation in the taxonomic structure of the microbiome given the data that we are able to analyze.

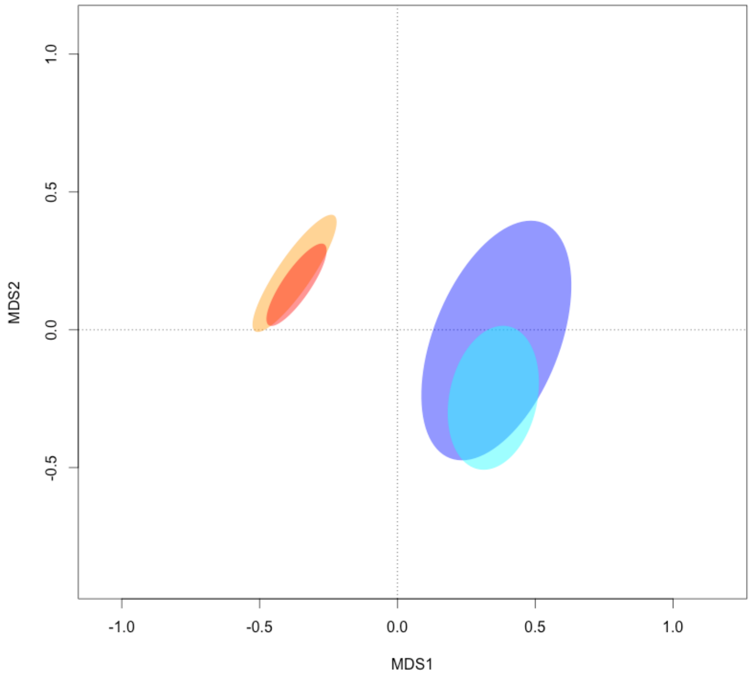

Figure S1. Gut microbiota difference between Hadza and Italians. PCA of Euclidean distances between genus-level gut microbiota profiles of Hadza and Italians. Ellipses include 95% confidence area based on the standard error of the weighted average of sample coordinates. Genera were filtered for those with >0.1% of relative abundance in at least 30% of subjects. Red, the original cohort of 27 Hadza from Schnorr et al. (2014); orange, the sub-cohort of 17 Hadza used in the present study; cyan, the original cohort of 16 Italian adults from Schnorr et al. (2014); blue, the sub-cohort of 12 Italian adults used in the present study. The two components explain 39% and 14% of the variance, respectively. A significant separation of the gut microbiota profiles by population was found within the original cohort from Schnorr et al. (2014) as well as in the smaller cohort of the present study ($P<1\times10^{-4}$, permutation test with pseudo F ratios).

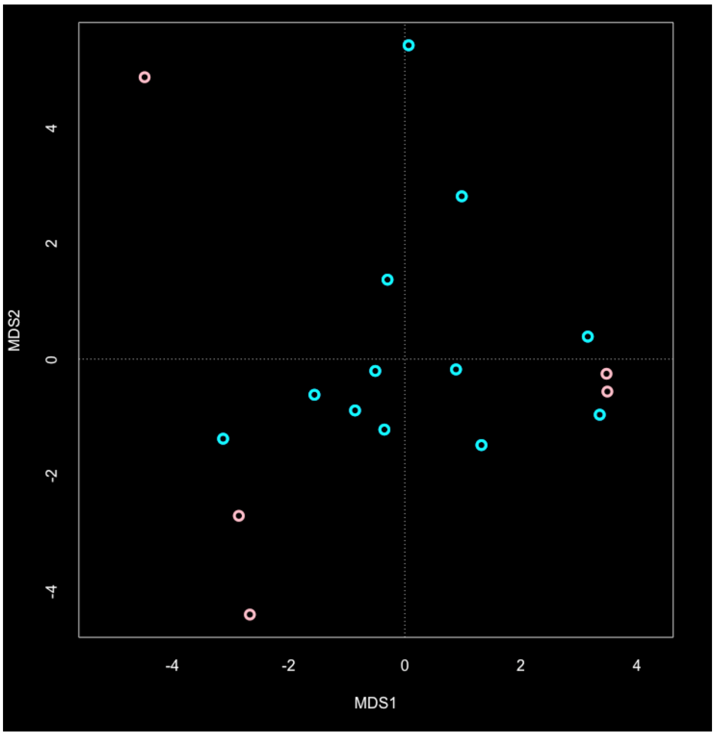

Figure S2. No sex difference in the fecal metabolome of the Hadza. PCA of Euclidean distances between the metabolic profiles of fecal extracts from the sub-cohort of 17 Hadza used in the present study. Only fecal metabolites significantly correlated (positively or negatively) with at least one genus of the gut microbiota within the overall dataset (*P*<0.05, Kendall tau correlation test) were considered. Pink, females; blue, males. *P*>0.05, permutation test with pseudo F ratios.

A

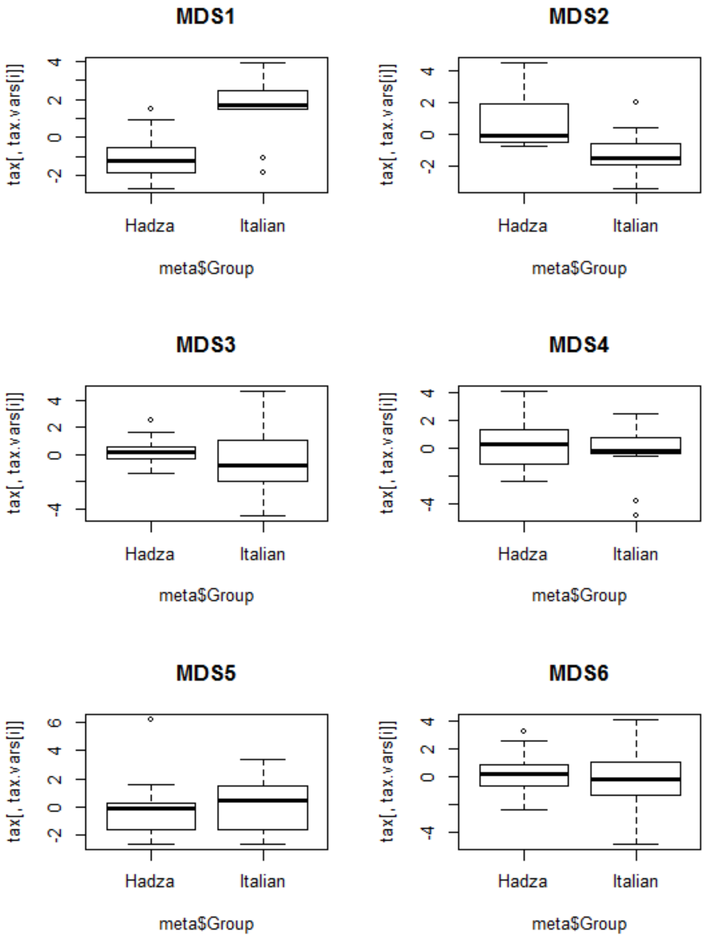
MDS1
MDS2
MDS3
MDS4
MDS5
MDS6
tax[, tax.vars[i]]
Hadza
Italian
meta$Group

B

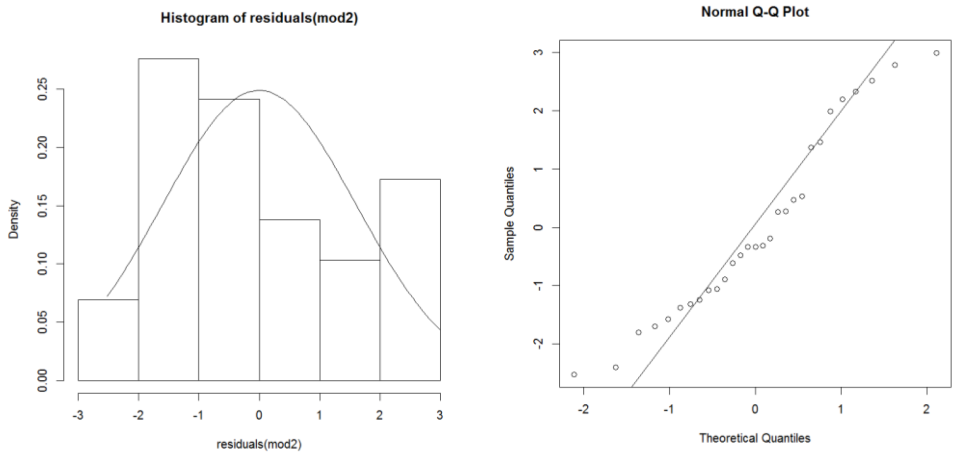
Histogram of residuals(mod2)
Density
residuals(mod2)
Normal Q-Q Plot
Sample Quantiles
Theoretical Quantiles

C

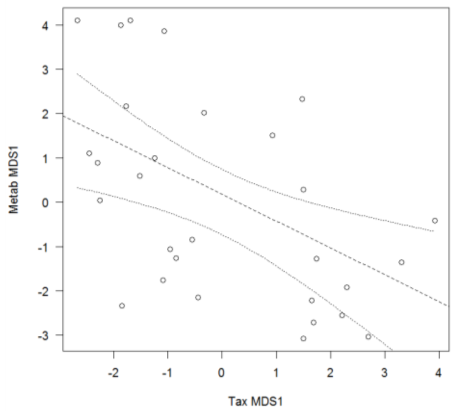

Figure S3. Multiple regression analysis to test the influence of age, sex and microbiome taxonomic composition on the metabolome profile from Hadza and Italians. A, Strong collinearity for MDS1 of the taxonomy PCA site scores and group assignment. **B,** Histogram of residuals and normal Q-Q plot evidence that the final model does not suffer from violation of assumptions of normality and homoscedasticity. **C,** Plot of the taxonomy MDS1 explanatory variable with the metabolome MDS1 response and 95% confidence intervals based on the model fit indicated an inverse relationship between the variance of these site scores.

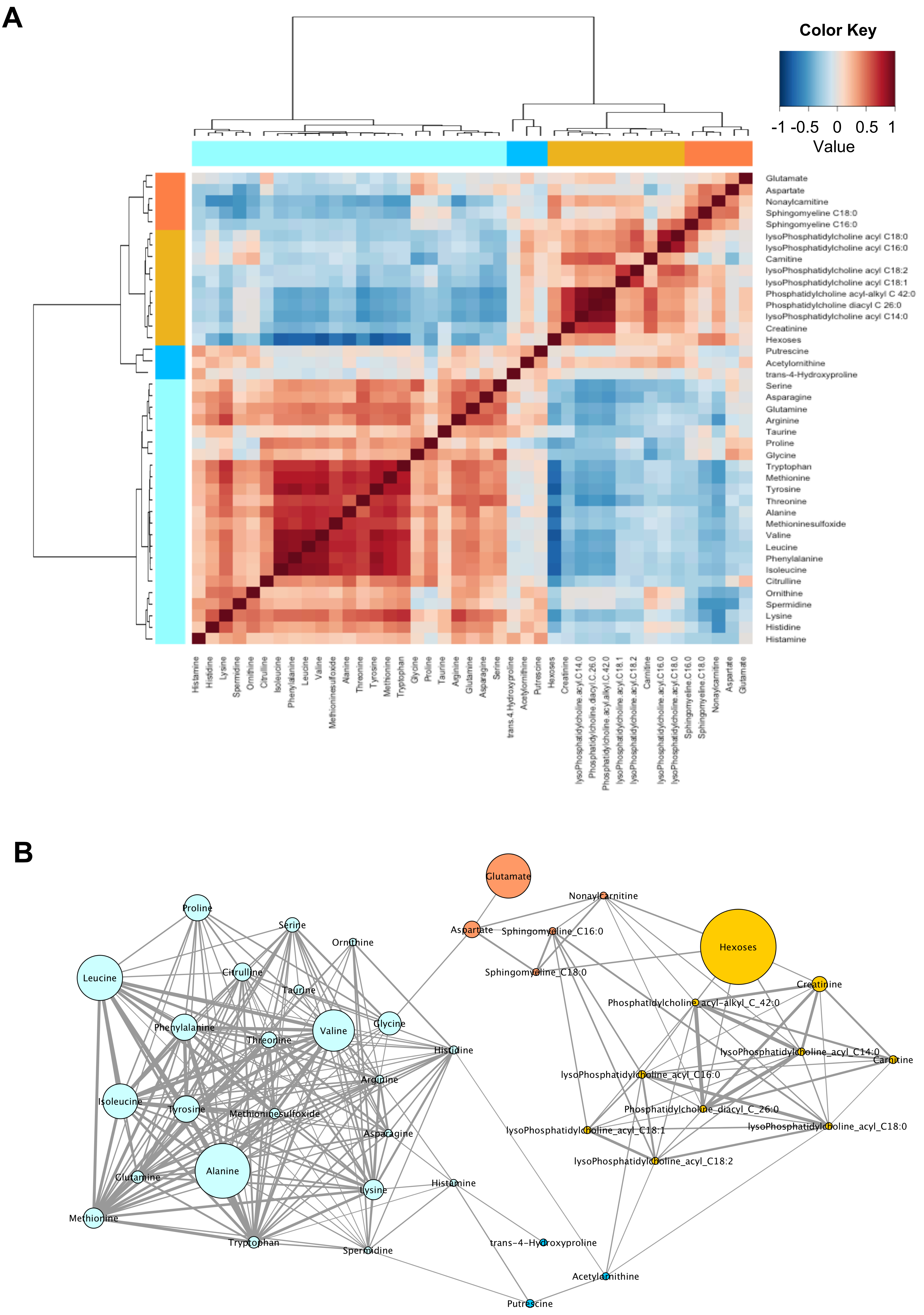
A
Color Key
-1 -0.5 0 0.5 1
Value
Glutamate
Aspartate
Nonaylcarnitine
Sphingomyeline C18:0
Sphingomyeline C16:0
lysoPhosphatidylcholine acyl C18:0
lysoPhosphatidylcholine acyl C16:0
Carnitine
lysoPhosphatidylcholine acyl C18:2
lysoPhosphatidylcholine acyl C18:1
Phosphatidylcholine acyl-alkyl C 42:0
Phosphatidylcholine diacyl C 26:0
lysoPhosphatidylcholine acyl C14:0
Creatinine
Hexoses
Putrescine
Acetylornithine
trans-4-Hydroxyproline
Serine
Asparagine
Glutamine
Arginine
Taurine
Proline
Glycine
Tryptophan
Methionine
Tyrosine
Threonine
Alanine
Methioninesulfoxide
Valine
Leucine
Phenylalanine
Isoleucine
Citrulline
Ornithine
Spermidine
Lysine
Histidine
Histamine
B
Glutamate
Nonaylcarnitine
Aspartate
Sphingomyeline_C16:0
Sphingomyeline_C18:0
Hexoses
Creatinine
Phosphatidylcholine_acyl-alkyl_C_42:0
lysoPhosphatidylcholine_acyl_C14:0
Carnitine
lysoPhosphatidylcholine_acyl_C16:0
Phosphatidylcholine_diacyl_C_26:0
lysoPhosphatidylcholine_acyl_C18:1
lysoPhosphatidylcholine_acyl_C18:0
lysoPhosphatidylcholine_acyl_C18:2
Proline
Serine
Ornithine
Leucine
Citrulline
Taurine
Phenylalanine
Valine
Glycine
Threonine
Histidine
Arginine
Isoleucine
Tyrosine
Methioninesulfoxide
Asparagine
Alanine
Glutamine
Lysine
Histamine
Methionine
Tryptophan
Spermidine
trans-4-Hydroxyproline
Acetylornithine
Putrescine

Figure S4. Assignment of metabolic co-abundance groups (CAGs). A, CAGs were defined by heat plot showing Kendall correlations between fecal metabolites clustered by Spearman correlation and Ward linkage. Colors are indicative of the four identified CAGs. **B**, Network plot showing correlations between the four identified CAGs. Circle size is proportional to the metabolite abundance. Connections between nodes represent positive and significant Kendall correlations between metabolites (P<0.05). Fecal metabolites were filtered for significant correlation (positive or negative) with at least one genus of the gut microbiota (P<0.05, Kendall tau correlation test), and for those with >0.05% of relative abundance in at least 20% of subjects.

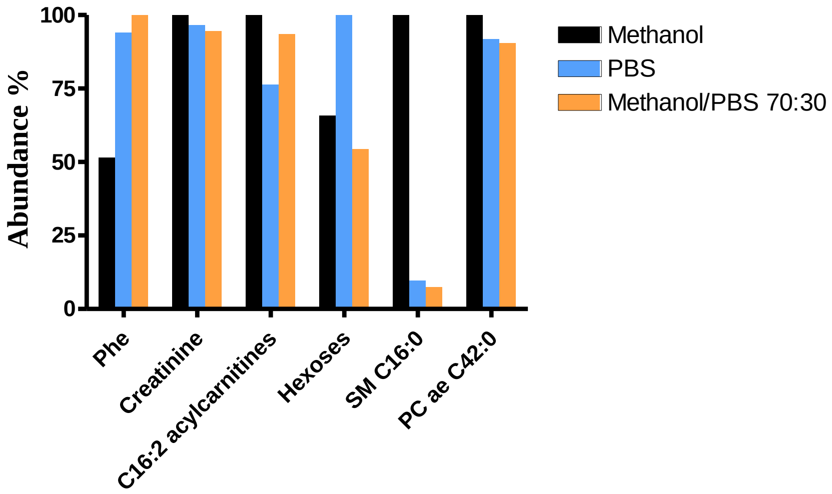

Figure S5. Evaluation of solvent extraction efficiency (n = 3). Methanol, phosphate buffer 10 nM, pH 7.5 (PBS) and a mixture of methanol/PBS 70:30 were evaluated for the extraction efficiency, expressed as normalized amount of tested compounds. Results for representative analytes of each metabolite class evidenced that there is no one solvent of choice for all analytes, however for the majority of the metabolites (acylcarnitines, phosphatidylcholines, lyso-phosphatidylcholines, sphingomyelins) methanol gave the best overall results (CV% < 25).

Table S1. Detailed list of all analyzed metabolites using the Absolute*IDQ* p180 Kit (BIOCRATES). For each metabolite, full and abbreviated names are provided, together with concentration values (mean ± SD) in fecal samples of Hadza and Italians. ND, not detected.

Metabolite abbreviation	Full name	Hadza (mean ± SD)	Italians (mean ± SD)
Acylcarnitines (nmol/g, n=40)			
C0	Carnitine	8.13 ± 3.11	19.87 ± 11.39
C2	Acetylcarnitine	0.39 ± 0.20	0.69 ± 0.33
C3	Propionylcarnitine	0.16 ± 0.06	0.27 ± 0.12
C3:1	Propenoylcarnitine	0.10 ± 0.04	0.16 ± 0.08
C3-OH	Hydroxypropionylcarnitine	0.19 ± 0.09	0.24 ± 0.12
C4	Butyrylcarnitine	0.15 ± 0.06	0.21 ± 0.10
C4:1	Butenylcarnitine	0.11 ± 0.04	0.16 ± 0.07
C4-OH (C3-DC)	Hydroxybutyrylcarnitine	0.32 ± 0.20	0.60 ± 0.36
C5	Valerylcarnitine	0.14 ± 0.06	0.21 ± 0.09
C5:1	Tiglylcarnitine	0.15 ± 0.08	0.24 ± 0.12
C5:1-DC	Glutaconylcarnitine	0.20 ± 0.09	0.17 ± 0.07
C5-DC (C6-OH)	Glutarylcarnitine (Hydroxyhexanoylcarnitine)	0.11 ± 0.04	0.16 ± 0.09
C5-M-DC	Methylglutarylcarnitine	1.10 ± 0.65	0.24 ± 0.10
C5-OH (C3-DC-M)	Hydroxyvalerylcarnitine (Methylmalonylcarnitine)	0.20 ± 0.09	0.38 ± 0.24
C6 (C4:1-DC)	Hexanoylcarnitine (Fumarylcarnitine)	0.18 ± 0.08	0.23 ± 0.13
C6:1	Hexenoylcarnitine	0.14 ± 0.06	0.18 ± 0.09
C7-DC	Pimelylcarnitine	0.15 ± 0.05	0.15 ± 0.07
C8	Octanoylcarnitine	1.10 ± 0.52	0.74 ± 0.32
C9	Nonaylcarnitine	3.71 ± 3.61	0.60 ± 0.32
C10	Decanoylcarnitine	1.54 ± 0.78	0.91 ± 0.43
C10:1	Decenoylcarnitine	0.47 ± 0.23	0.30 ± 0.14
C10:2	Decadienylcarnitine	0.36 ± 0.17	0.36 ± 0.23
C12	Dodecanoylcarnitine	0.64 ± 0.30	0.42 ± 0.28
C12:1	Dodecenoylcarnitine	0.43 ± 0.26	0.47 ± 0.33
C12-DC	Dodecanedioylcarnitine	1.36 ± 0.76	2.06 ± 2.20
C14	Tetradecanoylcarnitine	0.32 ± 0.29	0.54 ± 0.47
C14:1	Tetradecenoylcarnitine	0.15 ± 0.11	0.14 ± 0.06
C14:1-OH	Hydroxytetradecenoylcarnitine	0.30 ± 0.13	0.21 ± 0.17
C14:2	Tetradecadienylcarnitine	0.09 ± 0.03	0.09 ± 0.04
C14:2-OH	Hydroxytetradecadienylcarniti ne	0.20 ± 0.18	0.13 ± 0.07
C16	Hexadecanoylcarnitine	0.22 ± 0.07	0.36 ± 0.27
C16:1	Hexadecenoylcarnitine	0.25 ± 0.11	0.29 ± 0.16
C16:1-OH	Hydroxyhexadecenoylcarnitine	0.36 ± 0.18	0.20 ± 0.11
C16:2	Hexadecadienylcarnitine	0.11 ± 0.04	0.19 ± 0.09
C16:2-OH	Hydroxyhexadecadienylcarniti ne	0.23 ± 0.17	0.19 ± 0.09
C16-OH	Hydroxyhexadecanoylcarnitine	0.24 ± 0.10	0.26 ± 0.14

C18	Octadecanoylcarnitine	0.24 ± 0.13	0.64 ± 0.56
C18:1	Octadecenoylcarnitine	0.16 ± 0.10	0.36 ± 0.30
C18:1-OH	Hydroxyoctadecenoylcarnitine	0.32 ± 0.19	0.23 ± 0.10
C18:2	Octadecadienylcarnitine	0.10 ± 0.07	0.14 ± 0.09
Amino acids and biogenic amines (µmol/g, n=42)			
Ala	Alanine	0.60 ± 0.47	2.66 ± 1.87
Arg	Arginine	0.00 ± 0.00	0.06 ± 0.07
Asn	Asparagine	0.00 ± 0.00	0.03 ± 0.03
Asp	Aspartate	0.13 ± 0.12	0.09 ± 0.09
Cit	Citrulline	0.07 ± 0.05	0.34 ± 0.27
Gln	Glutamine	0.04 ± 0.03	0.16 ± 0.11
Glu	Glutamate	0.49 ± 0.39	0.88 ± 0.68
Gly	Glycine	0.15 ± 0.17	0.40 ± 0.29
His	Histidine	0.00 ± 0.00	0.06 ± 0.05
Ile	Isoleucine	0.16 ± 0.16	1.09 ± 0.65
Leu	Leucine	0.30 ± 0.29	1.78 ± 1.16
Lys	Lysine	0.04 ± 0.03	0.54 ± 0.41
Met	Methionine	0.06 ± 0.05	0.45 ± 0.29
Orn	Ornithine	0.00 ± 0.00	0.02 ± 0.02
Phe	Phenylalanine	0.12 ± 0.12	0.67 ± 0.41
Pro	Proline	0.18 ± 0.16	0.51 ± 0.43
Ser	Serine	0.05 ± 0.05	0.22 ± 0.17
Thr	Threonine	0.04 ± 0.03	0.32 ± 0.22
Trp	Tryptophan	0.02 ± 0.02	0.17 ± 0.11
Tyr	Tyrosine	0.11 ± 0.10	0.70 ± 0.42
Val	Valine	0.25 ± 0.23	1.44 ± 0.81
Ac-Orn	Acetylornithine	0.00 ± 0.00	0.01 ± 0.01
ADMA	Asymmetric dimethylarginine	0.00 ± 0.00	0.00 ± 0.00
SDMA	Symmetric dimethylarginine	0.00 ± 0.00	0.00 ± 0.00
alpha-AAA	alpha-Aminoadipic acid	0.00 ± 0.00	0.00 ± 0.00
Carnosine	Carnosine	0.00 ± 0.00	0.00 ± 0.00
Creatinine	Creatinine	0.08 ± 0.21	0.04 ± 0.05
Histamine	Histamine	0.00 ± 0.00	0.02 ± 0.03
Kynurenine	Kynurenine	0.00 ± 0.00	0.00 ± 0.00
Met-SO	Methioninesulfoxide	0.02 ± 0.01	0.11 ± 0.08
Nitro-Tyr	Nitrotyrosine	ND	ND
cis-OH-Pro	cis-4-Hydroxyproline	0.00 ± 0.00	0.00 ± 0.00
trans-OH-Pro	trans-4-Hydroxyproline	0.00 ± 0.00	0.01 ± 0.01
PEA	Phenylethylamine	0.01 ± 0.01	0.00 ± 0.00
Putrescine	Putrescine	0.01 ± 0.01	0.04 ± 0.07
Sarcosine	Sarcosine	0.00 ± 0.00	0.00 ± 0.00
Serotonin	Serotonin	0.00 ± 0.00	0.00 ± 0.00
Spermidine	Spermidine	0.00 ± 0.00	0.01 ± 0.02
Spermine	Spermine	0.00 ± 0.00	0.00 ± 0.00
Taurine	Taurine	0.01 ± 0.01	0.14 ± 0.29
Dopamine	Dopamine	0.00 ± 0.00	0.00 ± 0.00
DOPA	DOPA	0.00 ± 0.00	0.00 ± 0.00
Monosaccharides (µmol/g, n=1)			
H1	Sum of Hexoses	2.58 ± 1.32	2.62 ± 2.10

Sphingolipids (nmol/g, n=15)			
SM (OH) C14:1	Hydroxysphingomyelin with acyl residue sum C14:1	0.11 ± 0.22	0.15 ± 0.22
SM C16:0	Sphingomyelin with acyl residue sum C16:0	5.51 ± 7.84	4.34 ± 5.50
SM C16:1	Sphingomyelin with acyl residue sum C16:1	0.09 ± 0.10	0.10 ± 0.12
SM (OH) C16:1	Hydroxysphingomyelin with acyl residue sum C16:1	0.27 ± 0.40	0.24 ± 0.29
SM C18:0	Sphingomyelin with acyl residue sum C18:0	5.59 ± 8.68	2.72 ± 4.45
SM C18:1	Sphingomyelin with acyl residue sum C18:1	0.16 ± 0.23	0.11 ± 0.16
SM C20:2	Sphingomyelin with acyl residue sum C20:2	0.02 ± 0.01	0.02 ± 0.02
SM C22:3	Sphingomyelin with acyl residue sum C22:3	0.01 ± 0.01	0.01 ± 0.01
SM (OH) C22:1	Hydroxysphingomyelin with acyl residue sum C22:1	0.54 ± 0.95	0.58 ± 0.87
SM (OH) C22:2	Hydroxysphingomyelin with acyl residue sum C22:2	0.09 ± 0.16	0.09 ± 0.14
SM C24:0	Sphingomyelin with acyl residue sum C24:0	1.18 ± 1.93	1.01 ± 1.28
SM C24:1	Sphingomyelin with acyl residue sum C24:1	0.89 ± 1.84	0.92 ± 1.35
SM (OH) C24:1	Hydroxysphingomyelin with acyl residue sum C24:1	0.12 ± 0.20	0.11 ± 0.12
SM C26:0	Sphingomyelin with acyl residue sum C26:0	0.09 ± 0.09	0.05 ± 0.05
SM C26:1	Sphingomyelin with acyl residue sum C26:1	0.05 ± 0.07	0.04 ± 0.03
Glycerophospholipids (nmol/g, n=90)			
lysoPC a C14:0	Lysophosphatidylcholine with acyl residue C14:0	6.73 ± 3.47	6.73 ± 3.85
lysoPC a C16:0	Lysophosphatidylcholine with acyl residue C16:0	8.81 ± 6.35	12.42 ± 10.49
lysoPC a C16:1	Lysophosphatidylcholine with acyl residue C16:1	0.54 ± 0.63	0.57 ± 0.31
lysoPC a C17:0	Lysophosphatidylcholine with acyl residue C17:0	0.50 ± 0.34	0.38 ± 0.24
lysoPC a C18:0	Lysophosphatidylcholine with acyl residue C18:0	3.28 ± 3.90	3.29 ± 2.64
lysoPC a C18:1	Lysophosphatidylcholine with acyl residue C18:1	6.59 ± 9.25	4.90 ± 5.49
lysoPC a C18:2	Lysophosphatidylcholine with acyl residue C18:2	4.15 ± 5.33	3.00 ± 3.15
lysoPC a C20:3	Lysophosphatidylcholine with acyl residue C20:3	0.17 ± 0.15	0.13 ± 0.09
lysoPC a C20:4	Lysophosphatidylcholine with acyl residue C20:4	0.15 ± 0.13	0.19 ± 0.22

lysoPC a C24:0	Lysophosphatidylcholine with acyl residue C24:0	0.35 ± 0.17	0.30 ± 0.22
lysoPC a C26:0	Lysophosphatidylcholine with acyl residue C26:0	0.08 ± 0.04	0.10 ± 0.05
lysoPC a C26:1	Lysophosphatidylcholine with acyl residue C26:1	0.05 ± 0.03	0.07 ± 0.04
lysoPC a C28:0	Lysophosphatidylcholine with acyl residue C28:0	0.21 ± 0.11	0.22 ± 0.10
lysoPC a C28:1	Lysophosphatidylcholine with acyl residue C28:1	0.07 ± 0.04	0.06 ± 0.02
PC aa C24:0	Phosphatidylcholine with diacyl residue sum C24:0	0.11 ± 0.06	0.11 ± 0.06
PC aa C26:0	Phosphatidylcholine with diacyl residue sum C26:0	1.93 ± 1.09	2.05 ± 1.25
PC aa C28:1	Phosphatidylcholine with diacyl residue sum C28:1	0.19 ± 0.13	0.20 ± 0.14
PC aa C30:0	Phosphatidylcholine with diacyl residue sum C30:0	1.06 ± 1.14	0.77 ± 0.60
PC aa C30:2	Phosphatidylcholine with diacyl residue sum C30:2	0.01 ± 0.01	0.01 ± 0.01
PC aa C32:0	Phosphatidylcholine with diacyl residue sum C32:0	0.00 ± 0.00	0.00 ± 0.00
PC aa C32:1	Phosphatidylcholine with diacyl residue sum C32:1	0.18 ± 0.16	0.07 ± 0.07
PC aa C32:2	Phosphatidylcholine with diacyl residue sum C32:2	0.06 ± 0.05	0.03 ± 0.02
PC aa C32:3	Phosphatidylcholine with diacyl residue sum C32:3	0.03 ± 0.03	0.03 ± 0.02
PC aa C34:1	Phosphatidylcholine with diacyl residue sum C34:1	2.21 ± 1.94	0.65 ± 0.67
PC aa C34:2	Phosphatidylcholine with diacyl residue sum C34:2	1.26 ± 1.64	1.04 ± 2.64
PC aa C34:3	Phosphatidylcholine with diacyl residue sum C34:3	0.39 ± 0.47	0.12 ± 0.21
PC aa C34:4	Phosphatidylcholine with diacyl residue sum C34:4	0.05 ± 0.10	0.02 ± 0.01
PC aa C36:0	Phosphatidylcholine with diacyl residue sum C36:0	0.44 ± 0.27	0.36 ± 0.22
PC aa C36:1	Phosphatidylcholine with diacyl residue sum C36:1	0.88 ± 1.01	0.48 ± 0.51
PC aa C36:2	Phosphatidylcholine with diacyl residue sum C36:2	1.46 ± 2.13	0.84 ± 2.00
PC aa C36:3	Phosphatidylcholine with diacyl residue sum C36:3	1.07 ± 1.25	1.67 ± 5.26
PC aa C36:4	Phosphatidylcholine with diacyl residue sum C36:4	0.85 ± 1.04	1.26 ± 3.87
PC aa C36:5	Phosphatidylcholine with diacyl residue sum C36:5	0.26 ± 0.50	0.06 ± 0.07
PC aa C36:6	Phosphatidylcholine with diacyl residue sum C36:6	0.17 ± 0.35	0.03 ± 0.01

PC aa C38:0	Phosphatidylcholine with diacyl residue sum C38:0	0.26 ± 0.21	0.19 ± 0.17
PC aa C38:1	Phosphatidylcholine with diacyl residue sum C38:1	0.07 ± 0.08	0.02 ± 0.03
PC aa C38:3	Phosphatidylcholine with diacyl residue sum C38:3	0.22 ± 0.32	0.12 ± 0.10
PC aa C38:4	Phosphatidylcholine with diacyl residue sum C38:4	0.22 ± 0.39	0.06 ± 0.03
PC aa C38:5	Phosphatidylcholine with diacyl residue sum C38:5	0.09 ± 0.09	0.05 ± 0.02
PC aa C38:6	Phosphatidylcholine with diacyl residue sum C38:6	0.12 ± 0.11	0.07 ± 0.04
PC aa C40:1	Phosphatidylcholine with diacyl residue sum C40:1	0.66 ± 0.41	0.70 ± 0.46
PC aa C40:2	Phosphatidylcholine with diacyl residue sum C40:2	0.08 ± 0.04	0.09 ± 0.06
PC aa C40:3	Phosphatidylcholine with diacyl residue sum C40:3	0.03 ± 0.02	0.03 ± 0.01
PC aa C40:4	Phosphatidylcholine with diacyl residue sum C40:4	0.05 ± 0.03	0.04 ± 0.02
PC aa C40:5	Phosphatidylcholine with diacyl residue sum C40:5	0.01 ± 0.01	0.00 ± 0.01
PC aa C40:6	Phosphatidylcholine with diacyl residue sum C40:6	0.42 ± 0.22	0.42 ± 0.21
PC aa C42:0	Phosphatidylcholine with diacyl residue sum C42:0	0.10 ± 0.07	0.10 ± 0.06
PC aa C42:1	Phosphatidylcholine with diacyl residue sum C42:1	0.03 ± 0.01	0.04 ± 0.03
PC aa C42:2	Phosphatidylcholine with diacyl residue sum C42:2	0.09 ± 0.05	0.11 ± 0.07
PC aa C42:4	Phosphatidylcholine with diacyl residue sum C42:4	0.02 ± 0.01	0.03 ± 0.01
PC aa C42:5	Phosphatidylcholine with diacyl residue sum C42:5	0.04 ± 0.02	0.03 ±0.02
PC aa C42:6	Phosphatidylcholine with diacyl residue sum C42:6	0.46 ± 0.27	0.51 ± 0.38
PC ae C30:0	Phosphatidylcholine with acyl-alkyl residue sum C30:0	0.30 ± 0.15	0.27 ± 0.15
PC ae C30:1	Phosphatidylcholine with acyl-alkyl residue sum C30:1	0.05 ± 0.04	0.03 ± 0.02
PC ae C30:2	Phosphatidylcholine with acyl-alkyl residue sum C30:2	0.08 ± 0.03	0.07 ± 0.04
PC ae C32:1	Phosphatidylcholine with acyl-alkyl residue sum C32:1	0.11 ± 0.10	0.05 ± 0.05
PC ae C32:2	Phosphatidylcholine with acyl-alkyl residue sum C32:2	0.05 ± 0.04	0.05 ± 0.03
PC ae C34:0	Phosphatidylcholine with acyl-alkyl residue sum C34:0	0.69 ± 0.67	0.10 ± 0.08
PC ae C34:1	Phosphatidylcholine with acyl-alkyl residue sum C34:1	0.39 ± 0.34	0.15 ± 0.12

PC ae C34:2	Phosphatidylcholine with acyl-alkyl residue sum C34:2	0.19 ± 0.22	0.07 ± 0.06
PC ae C34:3	Phosphatidylcholine with acyl-alkyl residue sum C34:3	0.12 ± 0.14	0.06 ± 0.03
PC ae C36:0	Phosphatidylcholine with acyl-alkyl residue sum C36:0	0.34 ± 0.18	0.20 ± 0.13
PC ae C36:1	Phosphatidylcholine with acyl-alkyl residue sum C36:1	0.31 ± 0.25	0.17 ± 0.14
PC ae C36:2	Phosphatidylcholine with acyl-alkyl residue sum C36:2	0.23 ± 0.23	0.08 ± 0.05
PC ae C36:3	Phosphatidylcholine with acyl-alkyl residue sum C36:3	0.50 ± 1.53	0.04 ± 0.02
PC ae C36:4	Phosphatidylcholine with acyl-alkyl residue sum C36:4	0.13 ± 0.13	0.06 ± 0.04
PC ae C36:5	Phosphatidylcholine with acyl-alkyl residue sum C36:5	0.08 ± 0.06	0.06 ± 0.04
PC ae C38:0	Phosphatidylcholine with acyl-alkyl residue sum C38:0	0.33 ± 0.16	0.29 ± 0.17
PC ae C38:1	Phosphatidylcholine with acyl-alkyl residue sum C38:1	0.05 ± 0.05	0.04 ± 0.03
PC ae C38:2	Phosphatidylcholine with acyl-alkyl residue sum C38:2	0.06 ± 0.06	0.04 ± 0.06
PC ae C38:3	Phosphatidylcholine with acyl-alkyl residue sum C38:3	0.12 ± 0.22	0.06 ± 0.08
PC ae C38:4	Phosphatidylcholine with acyl-alkyl residue sum C38:4	0.09 ± 0.08	0.05 ± 0.03
PC ae C38:5	Phosphatidylcholine with acyl-alkyl residue sum C38:5	0.12 ± 0.10	0.07 ± 0.05
PC ae C38:6	Phosphatidylcholine with acyl-alkyl residue sum C38:6	0.13 ± 0.34	0.04 ± 0.03
PC ae C40:1	Phosphatidylcholine with acyl-alkyl residue sum C40:1	0.04 ± 0.02	0.05 ± 0.06
PC ae C40:2	Phosphatidylcholine with acyl-alkyl residue sum C40:2	0.05 ± 0.05	0.05 ± 0.04
PC ae C40:3	Phosphatidylcholine with acyl-alkyl residue sum C40:3	0.03 ± 0.02	0.02 ± 0.01
PC ae C40:4	Phosphatidylcholine with acyl-alkyl residue sum C40:4	0.14 ± 0.06	0.13 ± 0.07
PC ae C40:5	Phosphatidylcholine with acyl-alkyl residue sum C40:5	0.05 ± 0.03	0.04 ± 0.02
PC ae C40:6	Phosphatidylcholine with acyl-alkyl residue sum C40:6	0.06 ± 0.04	0.04 ± 0.02
PC ae C42:0	Phosphatidylcholine with acyl-alkyl residue sum C42:0	2.06 ± 1.10	2.17 ± 1.35
PC ae C42:1	Phosphatidylcholine with acyl-alkyl residue sum C42:1	0.20 ± 0.09	0.23 ± 0.14
PC ae C42:2	Phosphatidylcholine with acyl-alkyl residue sum C42:2	0.03 ± 0.02	0.03 ± 0.01
PC ae C42:3	Phosphatidylcholine with acyl-alkyl residue sum C42:3	0.03 ± 0.01	0.02 ± 0.01

PC ae C42:4	Phosphatidylcholine with acyl-alkyl residue sum C42:4	ND	ND
PC ae C42:5	Phosphatidylcholine with acyl-alkyl residue sum C42:5	1.10 ± 0.60	1.16 ± 0.72
PC ae C44:3	Phosphatidylcholine with acyl-alkyl residue sum C44:3	0.06 ± 0.03	0.06 ± 0.03
PC ae C44:4	Phosphatidylcholine with acyl-alkyl residue sum C44:4	0.14 ± 0.08	0.16 ± 0.10
PC ae C44:5	Phosphatidylcholine with acyl-alkyl residue sum C44:5	0.12 ± 0.07	0.13 ± 0.09
PC ae C44:6	Phosphatidylcholine with acyl-alkyl residue sum C44:6	0.09 ± 0.05	0.10 ± 0.06

Table S2. Results of the final model of the multiple regression analysis.

Coefficients	Estimate	Std. Error	t value
(Intercept)	0.95253	1.32649	0.718
taxMDS1	-0.60427	0.18935	-3.191
taxMDS2	0.24733	0.18996	1.302
taxMDS3	0.16316	0.19414	0.84
taxMDS4	0.27786	0.19423	1.431
taxMDS5	-0.17287	0.19338	-0.894
taxMDS6	-0.11806	0.19147	-0.617
sexM	0.76966	0.83091	0.926
age	-0.04201	0.0358	-1.173
